# LentiPro26: novel stable cell lines for constitutive lentiviral vector production

**DOI:** 10.1038/s41598-018-23593-y

**Published:** 2018-03-27

**Authors:** H. A. Tomás, A. F. Rodrigues, M. J. T. Carrondo, A. S. Coroadinha

**Affiliations:** 1 iBET – Instituto de Biologia Experimental e Tecnológica, Apartado 12, 2781-901 Oeiras, Portugal; 2 Instituto de Tecnologia Química e Biológica António Xavier, Universidade Nova de Lisboa, Av. da República, 2780-157 Oeiras, Portugal; 3 Faculdade de Ciências e Tecnologia, Universidade Nova de Lisboa, 2829-516 Monte da Caparica, Portugal

## Abstract

Lentiviral vectors (LVs) are excellent tools to promote gene transfer and stable gene expression. Their potential has been already demonstrated in gene therapy clinical trials for the treatment of diverse disorders. For large scale LV production, a stable producer system is desirable since it allows scalable and cost-effective viral productions, with increased reproducibility and safety. However, the development of stable systems has been challenging and time-consuming, being the selection of cells presenting high expression levels of Gag-Pro-Pol polyprotein and the cytotoxicity associated with some viral components, the main limitations. Hereby is described the establishment of a new LV producer cell line using a mutated less active viral protease to overcome potential cytotoxic limitations. The stable transfection of bicistronic expression cassettes with re-initiation of the translation mechanism enabled the generation of LentiPro26 packaging populations supporting high titers. Additionally, by skipping intermediate clone screening steps and performing only one final clone screening, it was possible to save time and generate LentiPro26-A59 cell line, that constitutively produces titers above 10^6^ TU.mL^−1^.day^−1^, in less than six months. This work constitutes a step forward towards the development of improved LV producer cell lines, aiming to efficiently supply the clinical expanding gene therapy applications.

## Introduction

Lentiviral vectors (LVs) are remarkable tools for gene transfer, both *in vivo* and *in vitro*, due to their ability to permanently integrate into the cell genome of dividing and non-dividing cells, sustaining long-term stable expression. Since the firsts LV developments in the early 1990s, LVs have been improved in terms of safety and efficacy^1^; noteworthy is the development of 3^rd^ generation packaging system^2^ and self-inactivating (SIN) vectors^3,4^. LVs have also been reported to exhibit lower genotoxicity when compared to their simpler counterparts, γ-retroviral vectors (γ-RVs)^5,6^. These reasons justify the growing number of LV based gene therapy studies entering clinical trials^7^, with promising results for the treatment of neurodegenerative or genetic disorders, infectious diseases, and more recently cancer immunotherapy^8,9^. Very recently, the United States Food and Drug Administration approved the first gene therapy treatment using LVs, the tisagenlecleucel (marketed as KYMRIAH™). This treatment uses a SIN-LV to modify autologous T cells and treat B cell acute lymphoblastic leukaemia.

The approval of tisagenlecleucel marks the clinical-to-market transition of LVs into the gene therapy field, anticipating the need for large-scale production of clinical grade LV preparations. Traditionally, the production of LVs has relied on transient co-transfection of HEK293T cells with four expression cassettes: (i) Gag-Pro-Pol, coding for the viral structural proteins and enzymes; (ii) Rev, encoding Rev accessory protein which plays an important role on viral genome nuclear exportation; (iii) Envelope, coding for the glycoproteins that interact with target cell receptors to mediate virus cell entry; and (iv) Vector genome, carrying the gene of interest to be packaged into produced viral particles. Transient LV productions are easy to perform at small scale. However, transient LV productions are difficult to scale-up. Additional disadvantages include the heavy costs of high quality transfectable DNA and transfection reagents, substantial batch-to-batch variability and short production periods^10^. In this context, a stable LV cell line constitutively producing high titer LVs is highly desirable. However, the generation of such cell lines is challenging and time consuming, requiring several transductions/transfections and selection steps, all of which combined with clonal isolation, amplification and screening, to find the best LV producer clones. In addition, the cytotoxicity of both HIV-1 protease and glycoprotein from vesicular stomatitis virus (VSV-G) envelope (the most used envelope to pseudotype LVs) has hampered the establishment of stable LV producer cell lines^1^.

To deal with the cytotoxicity issues, cell lines with inducible systems for the expression of viral components have been developed^10^. Yet, when those inducible cell lines are used, LV production is maintained only for a short period of time after induction and additional purification steps may be required to remove the inducible agents^10^. To date, only three cell lines have been reported constitutively expressing all the LV components (Gag-Pro-Pol, REV, Envelope and Vector genome). In all the cases, the selection of a clone with stable and high expression of Gag-Pro-Pol seems to be the major challenge, being difficult to develop a stable high titer LV producer cell line exclusively by traditional plasmid cell transfection. To overcome expression limitations, several strategies were developed using viral vectors to introduce the LV components into cell genomes, promoting their high expression and facilitating the establishment of clones producing high LV titers. The STAR derived cell lines^11^ were the first LV producer cell lines to be established. During its development, γ-RVs were used to integrate a *gag-pro-pol* codon-optimized and *rev* expression cassettes into the genome of HEK293T cells. The remaining LV components were introduced by plasmid cell transfection. The STAR establishment evidenced that is possible to develop a cell line constitutively supporting high LV productivities. However, the Long Terminal Repeats (LTRs) and packaging (Ψ) sequences of the γ-RVs used in STAR cell line development are present in the genome of the LV producer cells, which could promote the generation of replication competent lentivirus (RCL), raising safety concerns^12^. Some years later, the RD2-MolPack-Chim3 LV producer cell line was developed using a recombinant hybrid baculo-AAV vector to successfully integrate the *gag-pro-pol* and *rev* genes into the cell genome, avoiding the usage of γ-RVs^13^. The Tat and envelope genes were introduced using SIN-LVs to minimize possible safety concerns^14,15^. Posterior specific analysis attested the safety of the RD2-MolPack-Chim3 cell line^13^. More recently, in WinPac derived cell lines development, a different approach used γ-RVs to integrate a reporter expression cassette into the cells genome to identify a clone supporting high reporter expression levels^16^. Subsequently, this reporter expression cassette was replaced by a new one containing a codon-optimized LV g*ag-pro-pol* sequence, by means of Cre recombinase mediated cassette exchange (RMCE). The remaining LV components were introduced by the traditional plasmid cell transfection. The removal of most of γ-RV sequences during the cassette exchange event decreases the risk of RCL formation. Nevertheless, the usage of the RMCE required additional steps of clone isolation and screening, making cell line development even longer and more laborious.

All the three LV producer cell lines mentioned reflect the active demand for improved stable LV producer systems. Herein, is described an alternative methodology to accelerate the establishment of LV producer cell lines presenting high titers, exclusively by using chemical transfections followed antibiotic selection steps during the entire cell line development process.

## Results

### Transient LV productions using T26S mutated or wild type viral protease

**Figure 1:**
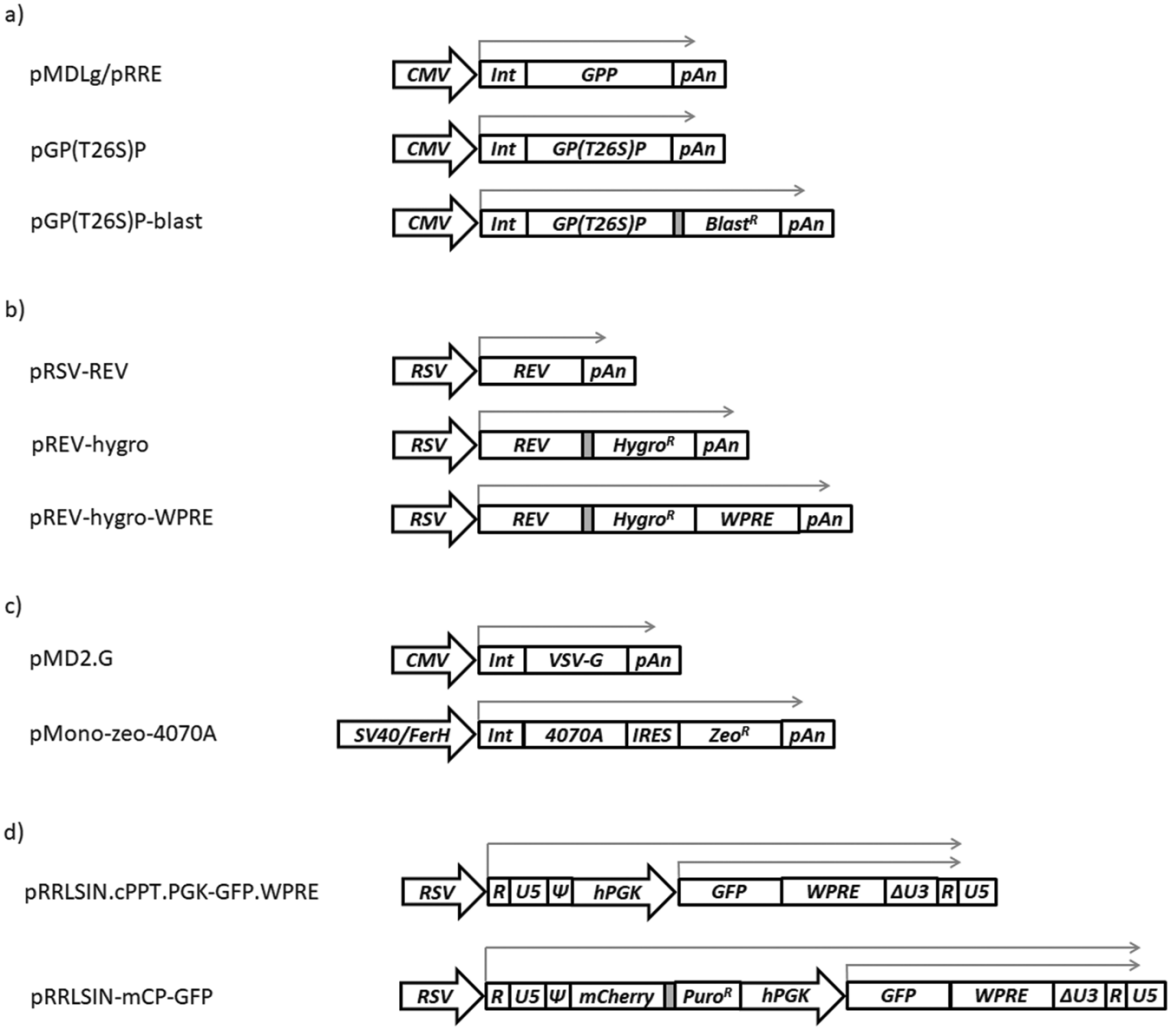
Schematic representation of the expression cassettes used in this work. (**a**) Gag-Pro-Pol expression cassettes. (**b**) Rev expression cassettes. (**c**) Envelope expression cassettes. (**d**) LV genome expression cassettes. Abbreviations: CMV, Cytomegalovirus promoter; Int, intron; GPP, gag-pro-pol sequence; pAn, polyA sequence; GP(T26S)P, gag-pro-pol with mutated T26S protease sequence; Blast^R^, blasticidin resistance gene; RSV, Rous Sarcoma Virus promoter; Hygro^R^, hygromycin resistance gene; WPRE, woodchuck hepatitis post-transcriptional regulatory element; VSV-G, glycoprotein G of the vesicular stomatitis virus; SV40/FerH, ferritin heavy chain (FerH) promoter fused with SV40 enhancer; IRES, internal ribosome entry site; Zeo^R^, zeocin resistance gene; Ψ, packaging signal sequence; hPGK, human phosphoglycerate kinase promoter; Puro^R^, puromycin resistance gene. The thinner grey arrows represent the mRNA transcripts. The filled grey boxes represent the spacer region that drives the re-initiation of the translation mechanism.

**Figure 2:**
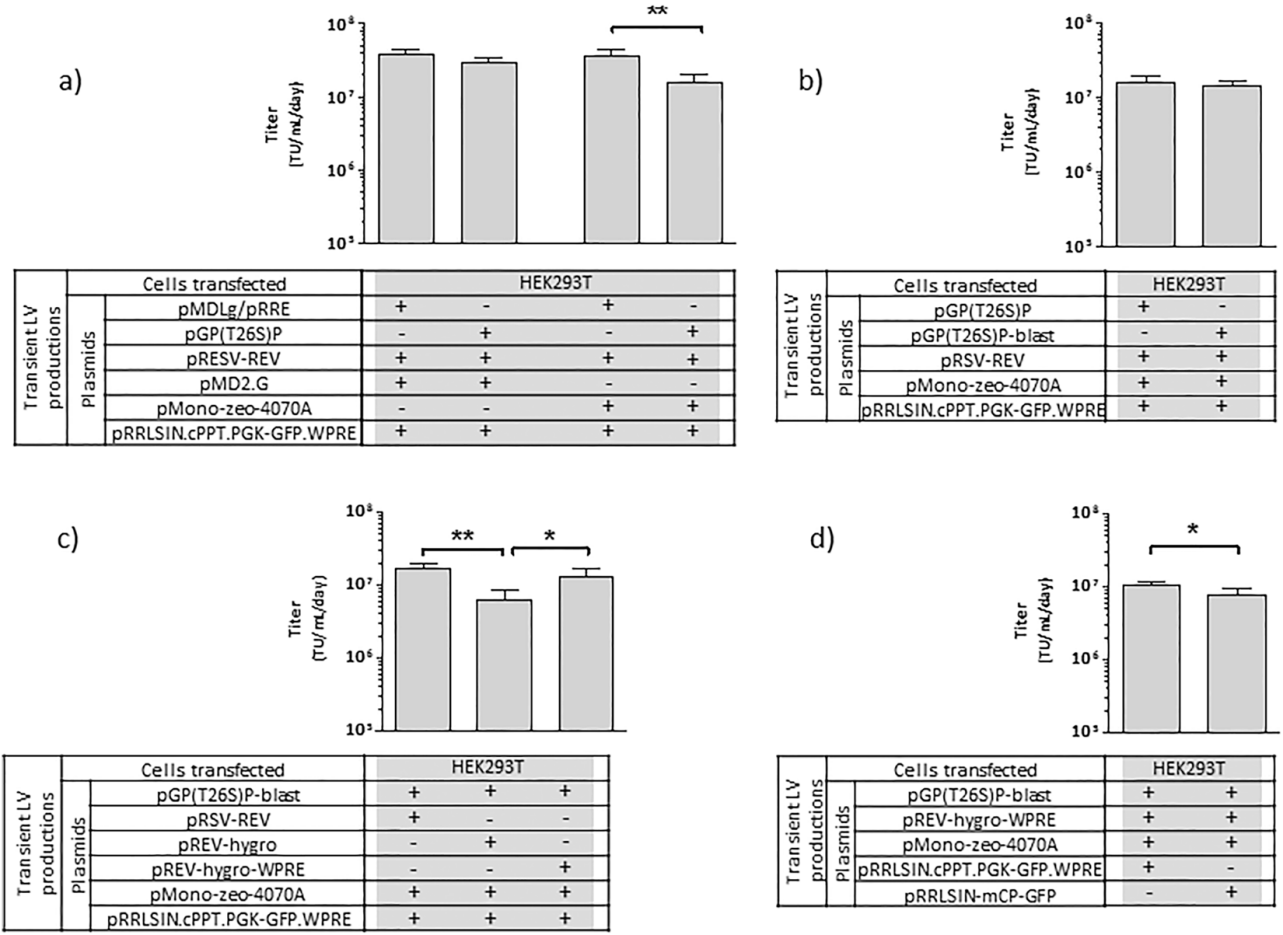
Transient LV productions by transfecting HEK293T cells. (**a**) Transient production titer comparison of LV pseudotyped with VSV-G or amphotropic envelope, using the wild type or the mutated T26S protease. (**b**) Transient LV production titer comparison of Gag-Pro(T26S)-Pol expression cassettes. (**c**) Transient LV production titer comparison of Rev expression cassettes. (**d**) Transient LV production titer comparison of vector genome expression cassettes. The (+) indicates presence and the (−) indicates absence of the respective plasmid in the transfection mix. Data shown represents the average ± standard deviation from 3 independent experiments. *(p < 0.05) and **(p < 0.005) given by one tailed unpaired t-test.

The T26S point mutation was performed in the viral protease of pMDLg/pRRE plasmid^2^, originating the pGP(T26S)P (Fig. 1a). This mutation was reported to decrease protease activity without affecting virus maturation and infectivity^17^, potentially leading to lower cytotoxicity when stably expressed. Ultimately, this could support higher expression levels of Gag-Pro(T26S)-Pol. The functionality of T26S protease was assessed by transient production of LVs pseudotyped with VSV-G or with amphotropic envelope (Fig. 2a). As a control, the wild type protease was also evaluated. No differences in infectious viral titers obtained were observed for LV productions with VSV-G envelope, whereas a 2-fold decrease on infectious LV titer was detected for viral production using the T26S mutated protease with amphotropic envelope. Titers above 10^7^ TU.mL^−1^.day^−1^ were achieved for all LV productions and the amphotropic envelope was used to proceed with stable cell line establishment.

### Expression cassettes construction for stable expression of LV components

To enable the selection of a population with high and stable expression of 3^rd^ generation LV packaging functions (Gag-Pro-Pol and Rev), the expression of a selectable marker was linked to the expression of each viral component. In the case of pGP(T26S)P plasmid, a blasticidin resistance gene (*bsr*) was introduced and coupled to the expression of *gag-pro(T26S)-pol* gene through a 81 bp spacer driving a re-initiation of translation mechanism^18,19^. This mechanism supports the selection of high expressing cells, and has been successfully used in the establishment of stable and high titer γ-retroviral vector producer cell lines^20–22^. This new pGP(T26S)-blast plasmid (Fig. 1a) was used in transient LV productions, presenting no differences in titers when compared to LV productions using pGP(T26S)P (Fig. 2b).

For stable Rev expression, the hygromycin B antibiotic resistance gene (*hph*) was inserted after *rev* coding sequence of the pRSV-REV plasmid^2^, originating the pREV-hygro (Fig. 1b); the expression of both *rev* and *hph* genes is also tightly linked through the re-initiation of the translation mechanism. A decrease in LV titer was observed when using pREV-hygro in transient LV productions (Fig. 2c). To note that the addition of *hph* gene to Rev expression cassette resulted in a 3-fold length increase of the Rev mRNA transcript, which could decrease mRNA stability and consequently reduce LV productivity. Thus, a woodchuck hepatitis post-transcriptional regulatory element (WPRE), reported to increase mRNA stability and to help the viral mRNAs nuclear exportation^23,24^, was inserted downstream of the *hph* gene. With this new plasmid, pREV-hygro-WPRE (Fig. 1b), viral titers similar to those of pRSV-REV were obtained (Fig. 2c).

The improved safety of self-inactivating (SIN) lentiviral vector genome is becoming a standard requirement in clinical grade LV productions^25–27^. For this reason, the SIN vector genome plasmid pRRLSIN.cPPT.PGK-GFP.WPRE (Fig. 1d) was used as vector backbone. Following the rational used with the other LV components, an antibiotic resistance marker was introduced into this vector genome expression cassette to promote the selection of cells expressing high levels of SIN-LV genome. In this case, the puromycin antibiotic resistance gene (*pac*) was cloned upstream of the PGK promoter to discriminate the LTR-RSV driven mRNA from the internal PGK promoter driven transcript. Moreover, the expression of puromycin resistance gene was coupled to the expression of a mCherry reporter gene through the re-initiation of translation mechanism, aiming to facilitate the isolation of clones with high vector genome expression. The functionality of this new pRRLSIN-mCP-GFP plasmid was evaluated by transient LV productions, presenting titers close to those obtained with pRRLSIN.cPPT.PGK-GFP.WPRE (Fig. 2d).

### Development of LentiPro26 packaging populations

**Figure 3:**
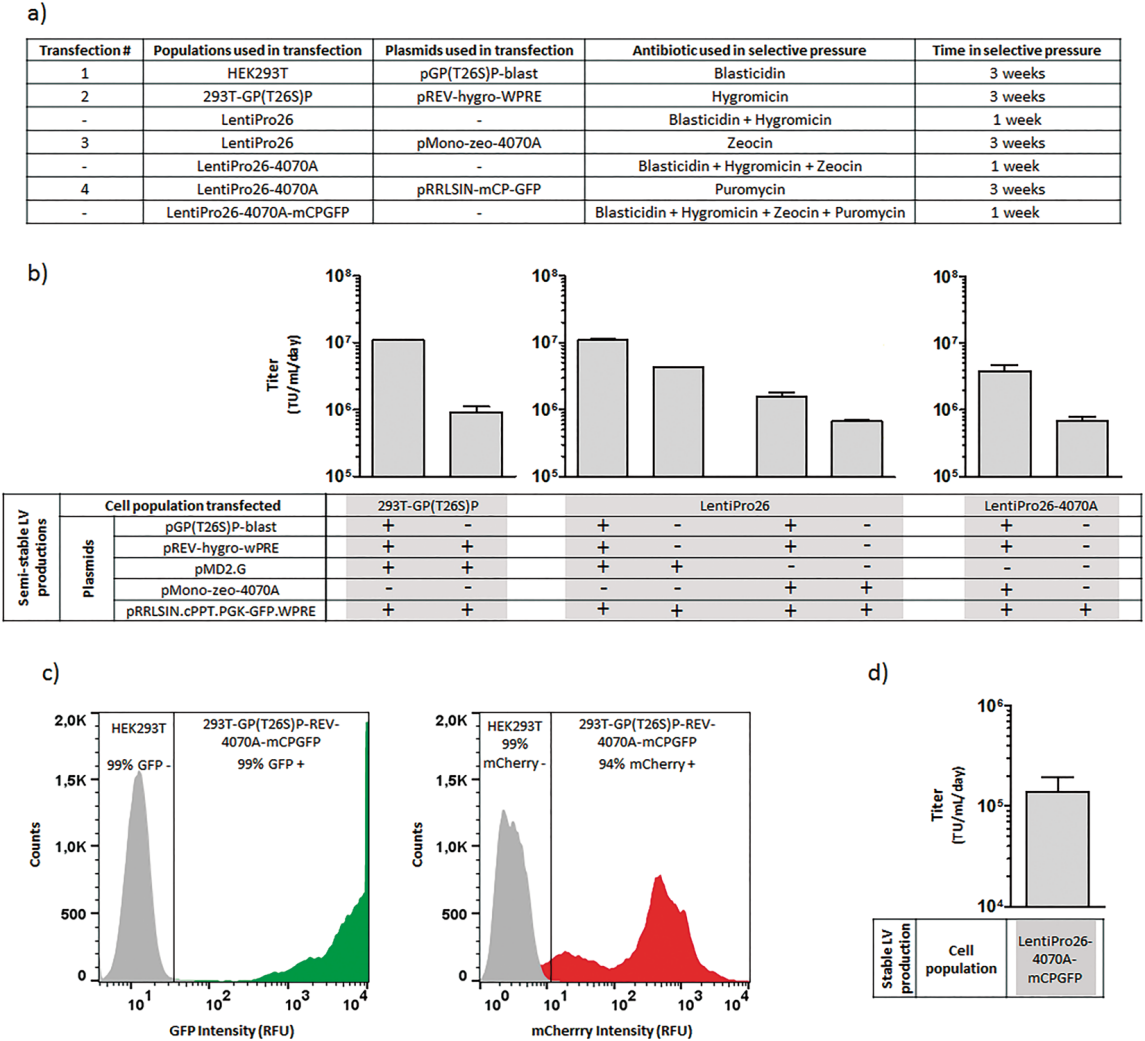
LV packaging and producer populations establishment and respective LV productions. (**a**) Development steps of the LentiPro26-4070A-mCPGFP population. (**b**) Titer of semi-stable LV productions for the 293T-GP(T26S)P, LentiPro26 and LentiPro26-4070A populations. The (+) indicates presence and the (−) indicates absence of the respective plasmid in the transfection mix. (**c**) Percentage of GFP or mCherry positive cells in LentiPro26-4070A-mCPGFP population (**d**) LV titer obtained in stable production with the population LentiPro26-4070A-mCPGFP. For all LV productions, the data shown represents the average ± standard deviation from 2 independent experiments.

The LV producer cell line development was initiated by stable polyethylenimine (PEI) transfection of HEK293T cells with the pGP(T26S)P-blast. After transfection, antibiotic selective pressure was applied (Fig. 3a) and a blasticidin resistant population, named 293T-GP(T26S)P, was selected. This population should consist of a pool of cells with different expression levels (low/medium/high) of Gag-Pro(T26S)-Pol. To confirm Gag-Pro(T26S)-Pol stable expression, the population was transiently transfected with the remaining LV components (Rev, VSV-G envelope and SIN vector genome), delivering an average titer of 9 × 10^5^ TU.mL^−1^.day^−1^ (Fig. 3b). As transfection control, the population was also transiently transfected with all LV components (Fig. 3b). In the latter case, the majority of the cells should contain several episomal copies of pGP(T26S)P-bl plasmid, increasing the yielded viral vector titers, 1 × 10^7^ TU.mL^−1^.day^−1^.

Following the Gag-Pro(T26S)-Pol expression cassette, the plasmid pREV-hygro-WPRE was stably transfected and selected by hygromycin selective pressure. An additional double selection step (blasticidin plus hygromycin) was performed to maximize the expression levels of both (Gag-Pro(T26S)-Pol and Rev) LV components (Fig. 3a). The resultant cell population, named LentiPro26, was evaluated through semi-stable LV productions. The titers obtained were 4 × 10^6^ TU.mL^−1^.day^−1^ and 7 × 10^5^ TU.mL^−1^.day^−1^ for LVs pseudotyped with VSV-G and amphotropic envelopes, respectively (Fig. 3b).

Moving forward with the cell line development, the LentiPro26 population was stably transfected with the plasmid pMONO-zeo-4070A that codes for the MLV amphotropic envelope (Fig. 1c). In this plasmid, the expression of the zeocin antibiotic resistance gene (*Sh ble*) is coupled to the envelope expression by the internal ribosome entry site (IRES) of Foot and Mouth Disease Virus^28^. Following the selection approach used above, the transfected cells were cultured with zeocin for three weeks. The resultant LentiPro26-4070A population was then subjected to an additional triple selection step (blasticidin, hygromycin and zeocin) for one week (Fig. 3a), and later evaluated for viral components stable expression through a semi-stable LV production (Fig. 3b). LentiPro26-4070A semi-stable LV production generated a titer of 7 × 10^5^ TU.mL^−1^.day^−1^.

### Establishment of a stable LV producer cell line

**Figure 4:**
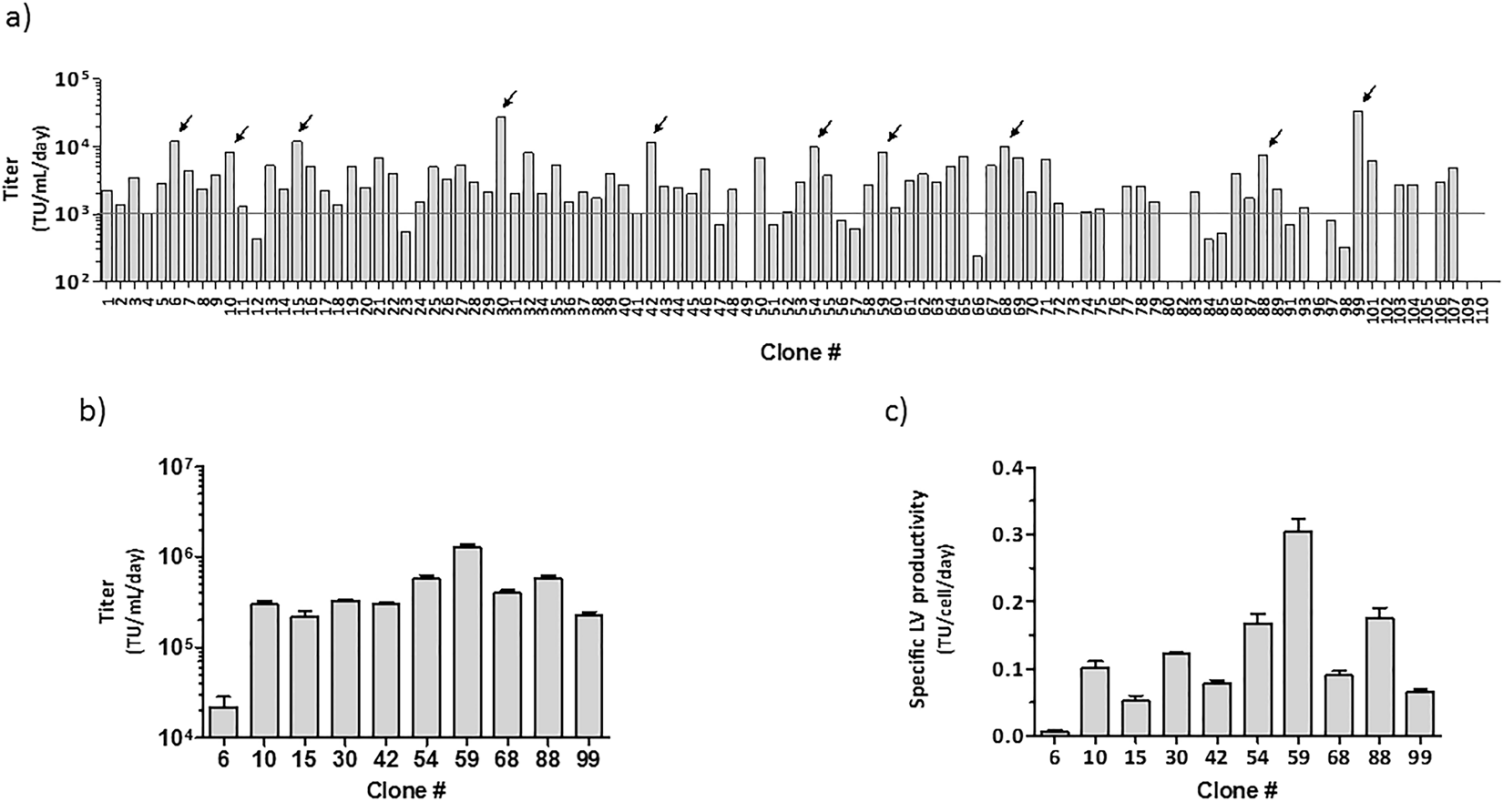
Stable LV production titers of the clones isolated from the LentiPro26-4070A-mCPGFP population. (**a**) Titer of the clones isolated by fluorescence-activated cell sorting and cultured in 2 cm^2^ plates. The arrows indicate the clones selected and the line represents the detection limit. The data shown represents the average of 2 replicates. (**b**) Titer of LV productions of the 10 best producer clones cultured in 25 cm^2^ plates. (**c**) Specific LV productivity of the 10 best producer clones cultured in 25 cm^2^ plates. The data shown represents the average ± standard deviation from 2 independent experiments.

To finally establish a cell population constitutively producing LVs, the LentiPro26-4070A population was transfected with the pRRLSIN-mCP-GFP plasmid and subjected to three weeks of puromycin selection followed by one week of selection with all antibiotics (Fig. 3a). The new resistant cell population presented a wide range of intensity levels of the reporter proteins, being 99% GFP positive and 94% mCherry positive (Fig. 3c). This population was called LentiPro26-4070A-mCPGFP and presented a stable LV production of 1.5 × 10^5^ TU.mL^−1^.day^−1^ (Fig. 3d). To find the best LV producer cell clones, 110 clones with high mCherry intensity fluorescence were isolated by fluorescence-activated cell sorting, and a small scale (2 cm^2^) stable LV production was performed (Fig. 4a). The top 10 LV producer clones were amplified and cultured in the presence of all antibiotics. A second stable LV production in 25 cm^2^ tissue culture flasks was performed to evaluate viral productivity. Clone 59, from now on designated LentiPro26-A59, presented the highest LV production (Fig. 4b,c), 1.6 × 10^6^ TU.mL^−1^.day^−1^, ten times higher than the parental LentiPro26-4070A-mCPGFP population. This clone was chosen for further characterization and process optimization.

### Influence of “medium volume/growth area” ratio in stable LV production

**Figure 5:**
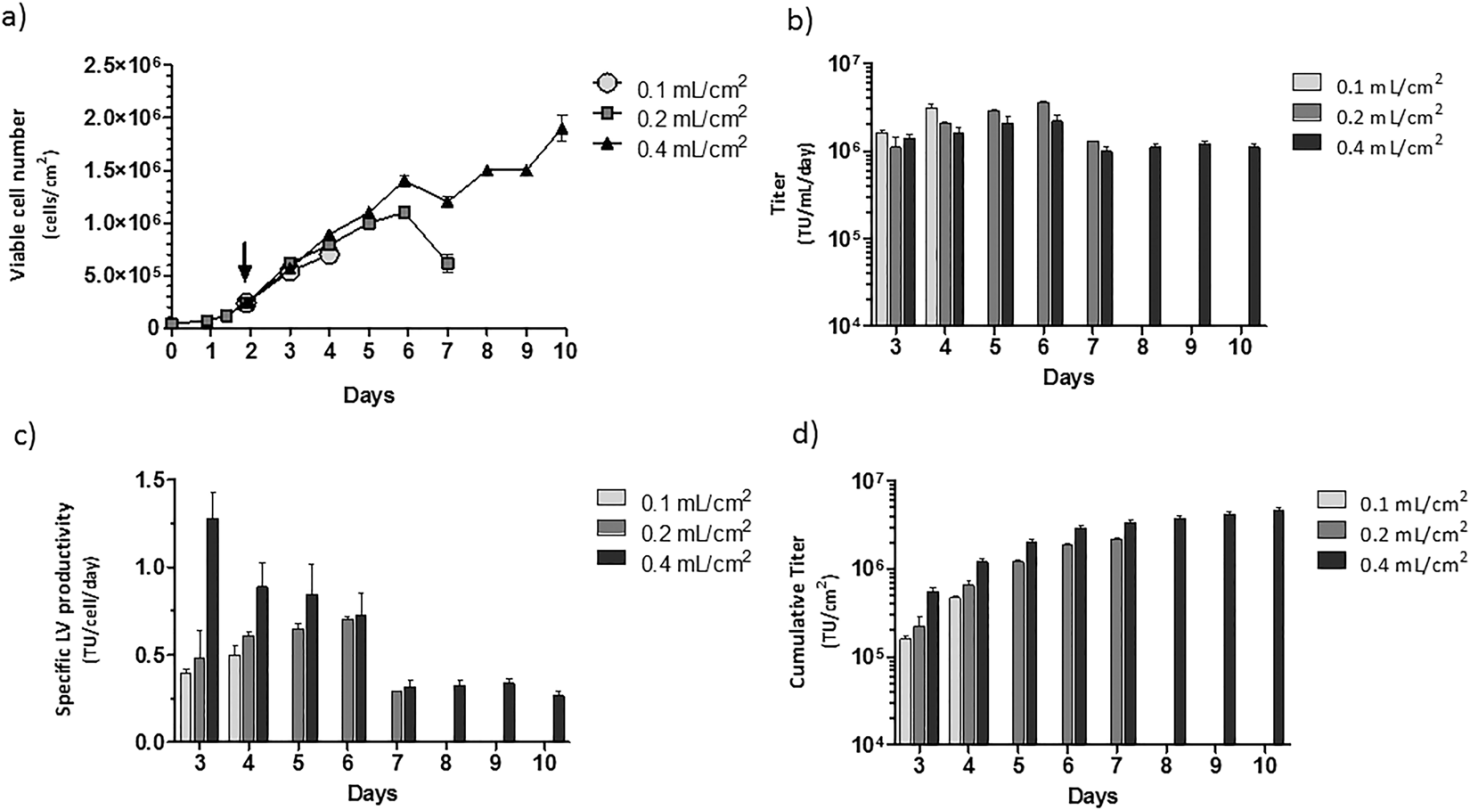
Optimization of LentiPro26-A59 viral productions to maximize viral productivity. (**a**) Viable cell concentration over time of a prolonged cell culture with increasing values of “medium volume/growth ratio” ratio. The black arrow represents the time point when medium exchange by fresh medium started to be performed every 24 hours. (**b**) Volumetric titer obtained in 24 hours production periods over the prolonged cell cultures. (**c**) Specific LV productivity obtained in 24 hours production periods over the prolonged cell cultures. (**d**) Cumulative titer of the cultures obtained when using increasing values of “medium volume/growth ratio” ratio. All the data shown represents the average ± standard deviation from 2 independent experiments.

Reducing the medium working volume in lentiviral production is a common strategy to increase supernatant viral concentration. Hence, the impact in cell growth and viral productivity, of medium volume per growth area ratio was assessed using 0.1, 0.2 and 0.4 mL/cm^2^ ratio values in stable LentiPro26-A59 viral productions. The ratio duplication from 0.1 to 0.2 mL/cm^2^ allowed three additional harvestings with titers above 1 × 10^6^ TU.mL^−1^.day^−1^. When the volume/area ratio was increased from 0.1 to 0.4 mL/cm^2^, the cell culture was further extended, allowing six additional harvestings, again presenting a titer average of 1 × 10^6^ TU.mL^−1^.day^−1^ (Fig. 5a,b). Notably, an increase in specific viral productivity was detected for the first 3 days of production (Fig. 5c) and a cumulative titer of 4.7 × 10^6^ TU/cm^2^ was obtained (Fig. 5d) at the end of culture, using the 0.4 mL/cm^2^ ratio.

### Impact of sodium butyrate in LentiPro26-A59 stable viral production

**Figure 6:**
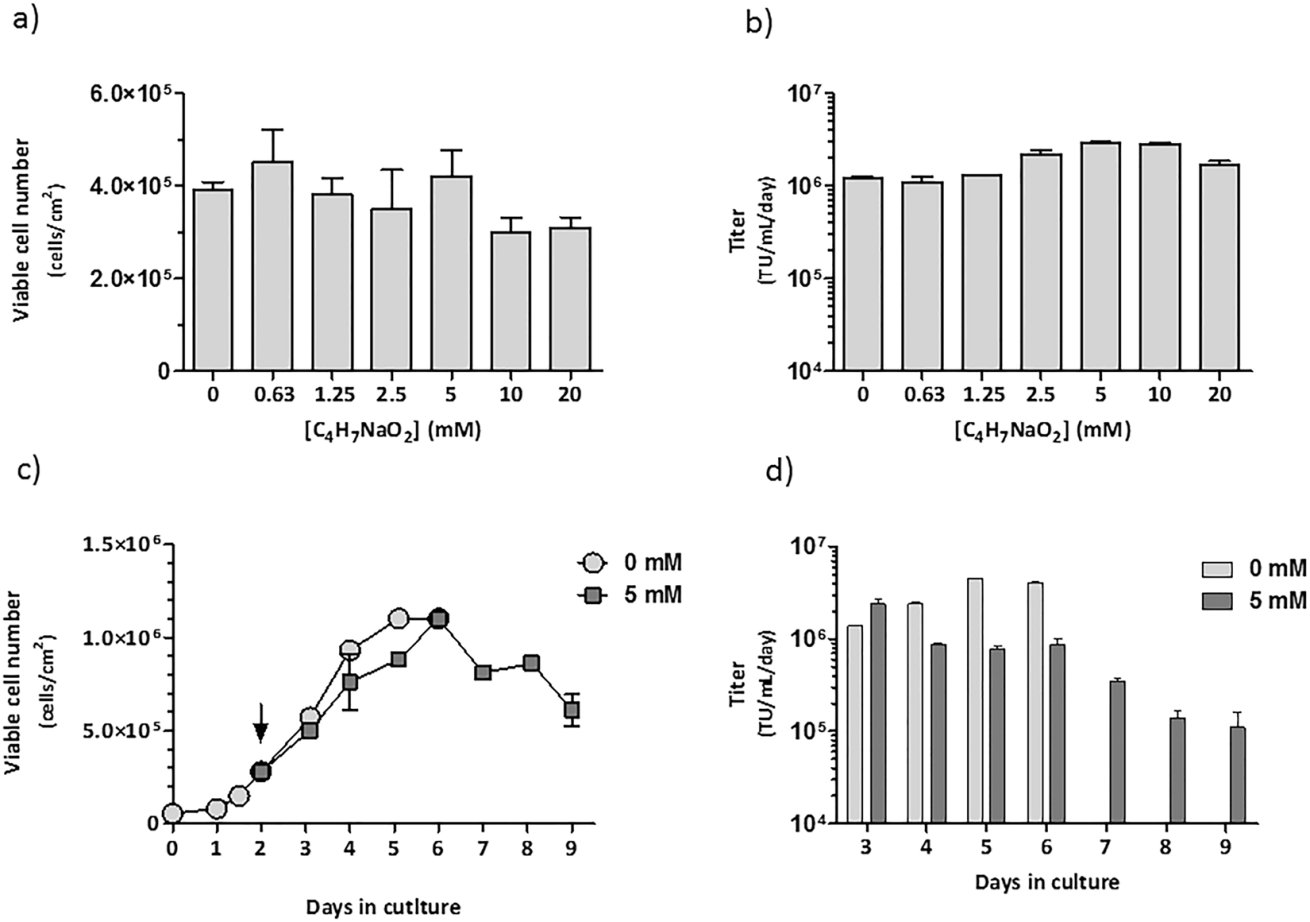
Influence of sodium butyrate in LentiPro26-A59 viral production. (**a**) Viable cell concentration of LentiPro26-A59 cell culture, 24 hours after medium exchange by fresh medium with increasing sodium butyrate concentrations. (**b**) Volumetric titers of stable LV productions performed using increasing sodium butyrate concentrations. (**c**) Viable cell concentration over a prolonged cell culture period in the presence or absence of sodium butyrate. The black arrow represents the time point when the medium started to be exchanged by fresh medium with 5 mM of sodium butyrate. (**d**) Volumetric titers obtained in 24 hours production periods over a prolonged cell culture period in the presence or absence of sodium butyrate. All data shown represents the average ± standard deviation from 2 independent experiments.

The addition of sodium butyrate to culture medium has been reported to increase LV titers for both adherent and suspension transient LV productions^29,30^. However, no data is available for stable LV production. Herein, we evaluated the impact of several sodium butyrate concentrations in 24 hours LentiPro26-A59 stable LV productions using 0.2 mL/cm^2^ volume/area ratio (Fig. 6a,b). A 3-fold increase in volumetric titer was obtained for LV productions with sodium butyrate concentrations of 5 mM and 10 mM; however, for 10 mM and 20 mM concentrations, a lower cell growth was observed. Thus, 5 mM concentration was chosen to evaluate the impact of sodium butyrate in cell growth and viral productivity for an extended cell culture period, exchanging medium every 24 hours. In the absence of sodium butyrate, cells reached the maximum concentration at day 5, only surviving until day 6. In the presence of sodium butyrate cells reached the same maximum concentration than without it, but one day later, surviving until day 9 (Fig. 6c). This extended survival could be related with lower acidification of the medium in the presence of sodium butyrate (detected during the culture through Phenol Red pH indicator). In contrast, in the absence of sodium butyrate a decrease in pH was observed. Regarding viral productivity, a 2-fold increase in LV titer was detected in the first 24 hours in contact with sodium butyrate (Fig. 6d), in line with the previous results (Fig. 6b). However, in the next days the titers decreased to values below 1 × 10^6^ TU.mL^−1^.day^−1^.

### Stability and scalability of LentiPro26-A59 LV production

**Figure 7:**
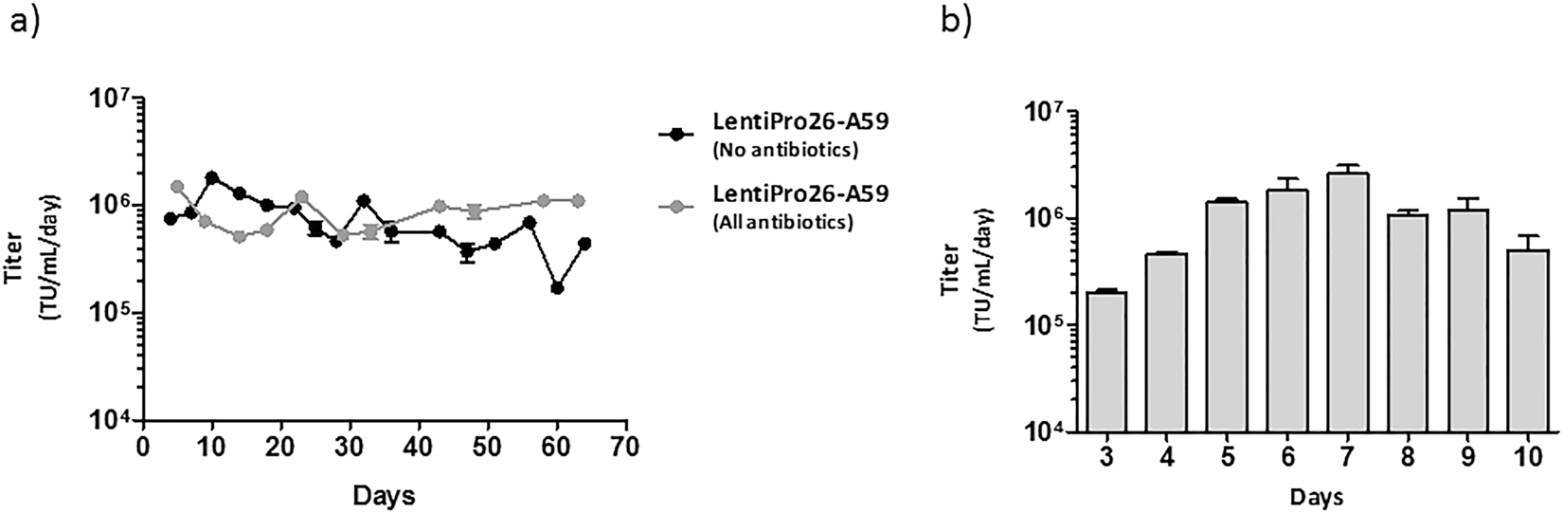
Stable LV productions of LentiPro26A-59 cell line. (**a**) LV production stability over time of LentiPro26-A59 cell line cultured for two months in the absence or presence of antibiotics. (**b**) Volumetric titers obtained in a HYPERFlask inoculated with LentiPro26A-59 cell line. Data is shown as average ± standard deviation of 2 replicates.

To evaluate the stability of LentiPro26-A59 viral productions, the cell line was cultured for 2 months in the presence or absence of the antibiotics used during the cell line development. A persistent production of about 1 × 10^6^ TU.mL^−1^.day^−1^ was observed during the first three weeks, in the absence of antibiotics. After that period, the titers started to decrease slowly over time reaching 4 × 10^5^ TU.mL^−1^.day^−1^ at day 65. In contrast, in the presence of all antibiotics, the titer was maintained near values of 1 × 10^6^ TU.mL^−1^.day^−1^ throughout all the culture period (Fig. 7a).

The reproducibility and scalability of LentiPro26-A59 viral productions was assessed by performing stable LV production using a HYPERFlask (Fig. 7b). Three days after inoculation, the HYPERFlask was 80% of confluent, being the supernatant harvested and exchanged for fresh medium every 24 hours during the following 7 days. A maximum titer of 2.2 × 10^6^ TU.mL^−1^.day^−1^ was achieved at day 7, being possible to perform up to 5 supernatant harvestings presenting volumetric productivities above 1 × 10^6^ TU.mL^−1^.day^−1^.

## Discussion

This work describes the development process of a HIV-1 derived LV producer cell line presenting stable viral productivities above 10^6^ TU.mL^−1^.day^−1^, exclusively based on chemical transfections of the viral constructs. The plasmids used herein were derived from those of the 3^rd^ generation system described in Dull *et al*.,^2^ keeping the *gag-pro-pol/rev* split cassette approach and maintaining the self-inactivating design of the vector genome. Additionally, with exception of the vector genome expression cassette, no further retroviral LTRs or Ψ sequences were used for increased safety standards.

To minimize potential cytotoxicity problems associated to the viral protease activity, a Gag-Pro-Pol construct harbouring the less active T26S mutated protease^17^ was used. This strategy may support the establishment of more robust cell lines, possibly allowing higher expression levels of *gag-pro-pol*, which has been reported as one of the main limitations for the development of high titer LV producer cell lines^11,13,16^.

Regarding the envelope glycoproteins, despite the higher titers for LVs pseudotyped with VSV-G (Fig. 2a), its cytotoxicity does not allow its constitutive expression^10^. The non-toxic MLV amphotropic envelope was used as proof-of-concept. Other non-toxic envelope glycoproteins may be constitutively expressed to pseudotype LVs such as the modified glycoproteins derived from gibbon ape leukemia virus (GaLV)^31^ or from feline endogenous retrovirus (RD114)^13,31^.

To steer selection of cells expressing high levels of LV components, the expression of different selectable markers was tightly coupled to the expression of the LV components using re-initiation of the translation mechanism or an IRES (Fig. 1). In the particular case of Gag-Pro-Pol expression cassette, the lower ribosomal translation rate of the Gag-Pro-Pol relatively to Gag polyprotein^32,33^, associated to the reduced translation efficiency of the gene after the spacer^18,19^ (selectable marker), drives a high stringent selection process and, consequently, promotes the selection of cells with high expression levels of the gene upstream the spacer (viral components).

The sequential PEI transfection and antibiotic selection of HEK293T cells, firstly with Gag-Pro(T26)-Pol and secondly with Rev expression cassettes, allowed the establishment of LentiPro26 packaging population. Semi-stable LV productions, by transfecting LentiPro26 cells with a SIN vector genome and the VSV-G or amphotropic envelope expression cassettes (Fig. 3b), generated titers similar or even higher to those obtained by semi-stable transfections of other reported packaging cell lines PK-7 (4 × 10^6^ TU.mL^−1^.day^−1^)^13^ and WinPack-57R10 (3 × 10^5^ TU.mL^−1^.day^−1^)^16^, evidencing the potential of plasmid transfection followed by antibiotic selection process for constitutive Gag-Pro(T26S)-Pol and Rev expression. Stable transfection of LentiPro26 population with amphotropic envelope allowed the selection of the packaging population LentiPro26-4070A which, when transiently transfected with the SIN vector genome plasmid, was able to produce 7 × 10^5^ TU.mL^−1^.day^−1^ (Fig. 3b). The same titer was achieved in previous semi-stable LV production using its parental population LentiPro26, indicating that stable expression of the amphotropic envelope in LentiPro26-4070A population was not limiting the viral production.

In addition to Gag-Pro-Pol, higher expression levels of the vector genome are likely to be important to sustain high titers. This is the case for stable γ-RV producer cell lines^22,34^. Aiming to improve the selection of cells with high expression levels of the vector genome, the expression of the selectable marker gene (puromycin) was coupled to the expression of a reporter gene (mCherry) under the control of the chimeric LTR-RSV promoter (Fig. 1d); the re-initiation of the translation mechanism was also used to assure a stringent selection of cells expressing high mCherry levels. With this new vector genome design, the mCherry expression is directly related to the expression of the vector genome transcript that will be encapsidated in the new LVs produced. Based on mCherry intensity, clones with high expression levels of the vector genome were isolated through fluorescence-activated cell sorting. This strategy evidenced to be successful since 9 out of the 10 top clones isolated delivered volumetric titers that appear to be superior to the parental population when compared across experiments (Figs 3d and 4b). The clone presenting higher LV productivity, named LentiPro26-A59, supports a titer of nearly 2 × 10^6^ TU.mL^−1^.day^−1^, producing about ten times more LVs than the parental population when compared across experiments (Figs 3d and 4b). Those LV packaging populations and LV producer cell line are the proof-of-concept that the strategy developed in this work could be used for the generation of LV producer cell lines in a shorter time. Additionally, demonstrates that is possible to generate relatively high titer LV producer cells only by traditional transfection followed by antibiotic selection process. The absence of retroviral LTRs and Ψ sequences in the Gag-Pro-Pol, Rev and envelope expression cassettes may also contribute for a safer cell line profile. Further approaches such as RMCE^35–37^, codon optimization^11,16^, concatemeric arrays^25^ or others could be used to establish new improved and safer high titer LV producer cells lines. Moreover, a full characterization of the LV components expression levels, the quality of LV preparations, the possibility of LV autotransduction, as well a careful analysis of RCL formation and LV genes mobilization are important to further characterize new LentiPro26-derived cell lines.

The possibility of performing several supernatant harvestings from a single culture or even continuous production is the major advantage of stable LV producer systems, reducing the LV decay due to its short half-lives^38,39^ and thus, making this a more cost-effective production process yielding higher infectivities. By manipulating the “volume/growth area” ratio values from 0.1 to 0.4 mL/cm^2^ of LentiPro26-A59 stable productions, we were able to increase cell productivity and extend the culture time, resulting in over 10-fold increase of the cumulative titer (Fig. 5d). We further improved the volumetric titer by adding sodium butyrate to the culture medium. Sodium butyrate is a well-known histone deacetylase inhibitor in mammalian cells; by preventing DNA compaction, it improves mRNA transcription and concomitantly, protein production^40^. Despite small increases in LV production during short production periods the presence of sodium butyrate impaired virus production in long term cultures (Fig. 6d). Similar observations were reported by Sakoda and colleagues^41^ where prolonged cell treatment with sodium butyrate reduced LV production. Other supplements with a more direct effect on viral productivity or a metabolic engineering approach can also be used to further increase LV production^42–44^.

The LV production stability over time and the reproducibility of the viral productions in larger scales are determinant factors for the successful commercial and clinical implementation of stable LV producer cell lines. LentiPro26-A59 cell line showed a consistent LV production for nearly one month in the absence of antibiotics and for at least two months in the presence of antibiotics. Furthermore, by inoculating a single HYPERFlask, a total 3.4 L of supernatant with a titer average of 1 × 10^6^ TU/mL was harvested. These results validate the reproducibility and scalability of the LentiPro26-A59 cell line, evidencing its potential to be adapted to continuous large-scale production systems.

Several silencing mechanisms of heterologous genes in mammalian cells impairing protein production are known^45,46^. A possible explanation for the LentiPro26-A59 viral productivity decrease over time could be the epigenetic silencing of the CMV promoter^47^ in Gag-Pro(T26S)-Pol expression cassette. Further analysis of the expression level of each LV component and the respective copy number in the cell genome over time may help to understand and find a solution to circumvent the loss of viral productivity over time.

The rational strategies used in this work substantially reduces the complexity and accelerates the generation process of potentially safer cell lines continuously producing LVs, making it similar to γ-RV producer cell line establishment process^48^. Further optimization of the codon-usage, the viral envelope glycoproteins and culture conditions might even increase LV productivities and safety, hence generating a competitive LV producer cell line to supply the demand of gene therapy clinical applications.

## Material and Methods

### Cell Culture

HEK 293 T cells (CRL-11268) obtained from the American Type Culture Collection (ATCC, Manassas, VA) were cultured in Dulbecco’s modified Eagle’s medium (DMEM) (Gibco, Life Technologies, Paisley, UK) supplemented with 10% (v/v) fetal bovine serum (FBS) (Gibco) and maintained at 37 °C in an incubator with a humidified atmosphere of 7% CO_2_ in air. During the selective pressure, antibiotics were added to the culture medium at appropriate concentrations (Invivogen, San Diego, USA) (Supplementary Table S1). Cell concentration and viability were assessed by trypan blue exclusion method.

### Plasmids

The primers and templates used in plasmid construction as well the cloning strategies are described in Supplementary Table S2.

The plasmid pMDLg/pRRE (Addgene #12251) codes for HIV-1 Gag-Pro-Pol under the control of a cytomegalovirus (CMV) promoter. The plasmid pRSV-REV (Addgene #12253) codes for HIV-1 Rev under the control of the rous sarcoma virus (RSV) U3 promoter. The plasmid pMD2.G (Addgene #12259) codes for envelope glycoprotein of the vesicular stomatitis virus (VSV-G) under the control of a CMV promoter. The plasmid pRRLIN.cPPT.PGK-GFP.WPRE (Addgene #12252) harbors a SIN vector genome under the control of a LTR-RSV chimeric promoter and drives the expression of GFP reporter from an internal promoter. All plasmids previously mentioned were kindly provided by Dr. Didier Trono through Addgene plasmid repository (Cambridge, MA).

The plasmid pGP(T26S)P is the result of a point mutation on pMDLg/pRRE viral protease sequence. With this point mutation, the protease 26^th^ amino acid is changed from a Threonine to a Serine as described in Konvalinka *et al*.^17^. The plasmid pGP(T26S)P-blast was generated by inserting *bsr* gene, amplified from pCEB plasmid^20^, into BspEI site of pGP(T26S)P plasmid.

The plasmid pREV-hygro is the ligation result of the reverse amplified pRSV-REV vector and the *hph* sequence, amplified from pSELECT-hygro-mcs (Invivogen). The WPRE sequence from pRRLIN.cPPT.PGK-GFP.WPRE was amplified and ligated to the reverse amplification of the vector pREV-hygro, originating the pREV-hygro-WPRE.

The plasmid pMONO-zeo-4070A was generated by cloning the 4070A sequence, amplified from the plasmid pENVA (kindly provided by Dr. Dagmar Wirth), into the vector pMONO-zeo-mcs (Invivogen) digested at the AvrII and AgeI sites.

Puromycin resistance gene (*pac*) was amplified from pSELECT-puro (Invivogen) and cloned into pRSV-REV, originating pREV-puro. The Rev sequence from pREV-Puro was then replaced by the mCherry sequence amplified from pRSET B^49^, originating the pRSV-mCherry-Puro. From this plasmid, the mCherry and *pac* sequences were amplified and cloned into EcoRV site of pRRLIN.cPPT.PGK-GFP.WPRE vector, originating the pRRLSIN-mCP-GFP.

### Lentiviral vector productions

#### Transient and semi-stable LV viral productions

Cells were seeded at 6 × 10^4^ cells/cm^2^ in tissue culture flasks. After 24 hours, cells were transfected using linear 25 KDa polyethyleneimine (PEI; Polysciences Inc, Hirschberg an der Bergstrasse, Germany) at a mass ratio of 1:1.5 (DNA:PEI), with the respective plasmids. The amount of each viral component *per* million of cells was: 2.5 µg of vector genome; 1 µg of Gag-Pro-Pol; 0.25 µg of Rev; 0.9 µg of envelope; 0 µg to 2.15 µg of a stuffer plasmid to ensure the same transfection conditions among all the semi-stable LV productions. Medium was replaced by 2.5 mL of fresh medium 24 hours after transfection. After another 24-hour period, the supernatant was harvested, clarified at 0.45 µm and stored at −80 °C.

#### Stable viral productions

Cells were seeded at 6 × 10^4^ cells/cm^2^ in tissue culture flasks. After 48 hours, medium was replaced by 2.5 mL or an indicated specific volume of fresh medium. After 24 hours the supernatant was harvested. When indicated, a specific volume of fresh medium was added again to the tissue culture flasks, the supernatant being harvested 24 hours later. All the supernatants were clarified at 0.45 µm and stored at −80 °C, upon harvesting.

### LV titration

The functional LV titers were determined by flow cytometric analysis for GFP expression of transduced HEK293T cells. Briefly, HEK293T cells were seeded at 7 × 10^4^ cells/cm^2^ in 24-well plates. After 24 hours, the cell supernatant was replaced by 0.2 mL of supernatant dilutions in fresh DMEM with 10% (v/v) FBS and 8 µg/mL of polybrene (Sigma, St Louis, MO, USA). The plates were centrifuged at 25 °C for 2 hours at 1200 g (spin-inoculation). After centrifugation, 0.6 mL of fresh DMEM with 10% (v/v) FBS was added to each well and the plates were incubated at 37 °C in an incubator with a humidified atmosphere of 7% CO_2_ in air. Cells were harvested and analyzed for GFP fluorescence by flow cytometry (CyFlow-space, Partec Gmbh, Munster, Germany) 48 hours after spin-inoculation. The number of LV transducing units per volume (TU/mL) was determined by the equation:

$${\rm{Titer}}(\frac{{\rm{TU}}}{{\rm{mL}}})=\frac{ \% \,{\rm{of}}\,{\rm{GFP}}\,{\rm{positive}}\,{\rm{cells}}\div100}{{\rm{volume}}\,{\rm{of}}\,{\rm{transduction}}}\times {\rm{dilution}}\,{\rm{factor}}\times {\rm{no}}\,{\rm{of}}\,{\rm{cells}}\,{\rm{at}}\,{\rm{the}}\,{\rm{infection}}\,{\rm{time}}$$

### Fluorescence-activated cell sorting

Cells were analysed and isolated by fluorescence activated cell sorting using MoFlo (Beckman Coulter, Fort Collins, Colorado, USA). The isolated clones were expanded in 96-well plates with DMEM with 20% (v/v) FBS.

### Clone screening

Two weeks after clone isolation, the single colonies were moved to 24 well-plates and cultured for two weeks in DMEM with 10% (v/v) FBS containing all antibiotics. When the clones reached 80% of confluence, the medium in each well was replaced by 0.25 mL of fresh medium with no antibiotics. After 24 hours 0.15 mL of supernatant was harvested and titrated for LVs.

## Electronic supplementary material

Supplementary Information
