# Supplementary material for: LentiPro26: novel stable cell lines for constitutive lentiviral vector production
Source: Sci Rep. 2018 Mar 27;8:5271. doi: 10.1038/s41598-018-23593-y (PMC5869598; doi:10.1038/s41598-018-23593-y)
Supplement: Supplementary file 1 — Supplementary Information [file 41598_2018_23593_MOESM1_ESM.pdf]

Supplementary information

Title

LentiPro26: novel stable cell lines for constitutive lentiviral vector production

Authors and affiliations

H.A. Tomás[1,2], A.F. Rodrigues[1,2], M.J.T. Carrondo[1,2,3], A.S. Coroadinha[1,2*]

[1] iBET – Instituto de Biologia Experimental e Tecnológica, Apartado 12, 2781-901 Oeiras, Portugal

[2] Instituto de Tecnologia Química e Biológica António Xavier, Universidade Nova de Lisboa, Av. da República, 2780-157 Oeiras, Portugal

[3] Faculdade de Ciências e Tecnologia, Universidade Nova de Lisboa, 2829-516 Monte da Caparica, Portugal

* Corresponding author

Supplementary information

Supplementary Table I: Antibiotic concentrations used to culture the cells.

**Antibiotics**	**Working concentration (µg/mL)**
Blasticidin	20
Hygromycin B Gold	150
Zeocin™	150
Puromycin	0.5

Supplementary Table II: Primers to amplify the fragments used in the plasmid constructions.

Final construct	Vector template	Primers used in vector amplification	Insert template	Primers used for insert amplification	Ligation reaction
pGP(T26S)P	pMDLg/pRRE	FW:TATTAGATTCTGGAGCAGATGATACAGTATTAGAAGA<br><br>RV:CTGCTCCAGAATCTAATAGAGCTTCCTTTAATT	-	-	GeneArt Mutagenesis Kit (Life Technologies, USA)
pGP(T26S)P-blast	pGP(T26S)P	-	pCEB	FW:CACATGGAATTCCGGATCCCCTTAATTCTTCTGATGCTC<br><br>RV:CTGCGGCCGCTCCGGATTAATTTCGGGTATATTTGAGTGGA	In-Fusion ligation (Clontech Laboratories, Inc.)
pREV-hygro	pRSV-REV	FW:GTAGCTGAGGGGACAGATAGGGTTA<br><br>RV:GTGGCATTGAGCAAGCTAACAGC	pSELECT-hygro-mcs	FW:CTTGCTCAATGCCACTCTAGCGAATTCTCGACT<br><br>RV:TGTCCCCTCAGCTACATCTGACGGTTCACTAAACG	In-Fusion ligation
pREV-hygro-WPRE	pREV-hygro	FW:AGCTGTAGCTGAGGGGACAGATA<br><br>RV:TTTAAGCTCCAGGCTTCCTTGT	pRRLSIN.cPPT.PGK-GFP.WPRE	FW:AGCCTGGAGCTTAAATCGACAATCAACCTCTGGATTACA<br><br>RV:CCCTCAGCTACAGCTAAGTCATTGGTCTTAAAGGTACCGA-	In-Fusion ligation
pMONO-zeo-4070A	pMono-zeo-mcs	-	pENVA	FW:ATGAACCGGTTTTTGTCTTTTATTTCAGGTC<br><br>RV:TAGTCCTAGGTACATAAGCGGATAACGGAT	T4 DNA ligase (New England Biolabs Inc, Ipswich, Massachusetts, USA)
pRRLSIN-mCP-GFP	pRRLSIN.cPPT.PGK-GFP.WPRE	-	pRSV-mCherry-Puro	FW:CTCGAGAAGCTTGATATCGCCGCCATGGTGAGCAAGGGCGAGGAGGATA<br><br>RV:CCGTGGGAATTCGATATCTTAAGCTCCAGGCTTCCTTGTCATG	In-Fusion ligation
